# 20.2 ANALYZING THE MOLECULAR EFFECTS OF LARGE NEUROPSYCHIATRIC CNVS WITH IPSC BASED NEURONAL TISSUE CULTURE MODELS

**DOI:** 10.1093/schbul/sby014.081

**Published:** 2018-04-01

**Authors:** Alexander Urban

**Affiliations:** Stanford University

## Abstract

**Background:**

Several large copy number variants (CNVs) in the genomic sequence are strongly associated with schizophrenia. These loci are important objects of study in their own right as well as enticing points of entry for the better understanding of the molecular etiology of schizophrenia. However, most of the schizophrenia-associated large CNVs are larger than one million base pairs and affect up to several dozen genes, presenting a complex challenge for research aiming to determine how these sequence variants are connected on the molecular level to the phenotype.

**Methods:**

We have established iPSC based tissue culture models for three of the major schizophrenia associated large CNVs, on chromosomes 22q11 (deletion), 15q13 (deletion) and 16p11 (deletion or duplication). We create neuronal cells with the defined genotypes using either direct induction into the neuronal state (induced neurons, iNs), by slower differentiation via neuronal precursor cells (NPCs) or by generating 3D cultures of cortical spheroids. We then assay the molecular effects of the large CNVs along the trajectory of differentiation by using RNA-Seq (transcriptome), ATAC-Seq (chromatin state) and SeqCap-Epi (DNA-methylation patterns). We also carry out single-cell RNA-Seq analysis using the drop-Seq approach.

**Results:**

We detect common effects across the large CNVs as well as locus-specific phenomena. For the most part genes within the CNV boundaries will change their expression patterns in concordance with their new copy number, with notable exceptions. Transcriptome-wide there is a network effect where several hundred genes are differentially expressed, including genes already identified as candidate genes for schizophrenia. Epigenomic states are affected, again most often not only in or nearby the boundaries of the large CNVs but epigenome-wide. Integrative analysis across the layers of molecular signals shows partial concordance as well as a degree of changes in signal being ‘offset’ between the levels, potentially owing to the dynamic differentiation state of the model system.

**Discussion:**

Neuronal tissue culture models based on iPSCs with defined large CNVs strongly associated with Schizophrenia allow for an analysis of the effects of such structural genomic sequence changes in disease-relevant cellular differentiation states. Application of cutting edge genomics and epigenomics assays and integrative data analysis reveals incomplete transcriptional dosage compensation of the genes within the large CNVs as well as transcriptome-wide network effects. Furthermore, there are epigenomic effects in the form of altered chromatin states that may to some extent mediate the gene expression changes. Differences between the large CNV loci as well as potential points of convergence will be discussed.

